# Inlet Effect Caused by Multichannel Structure for Molecular Electronic Transducer Based on a Turbulent-Laminar Flow Model

**DOI:** 10.3390/s20072154

**Published:** 2020-04-10

**Authors:** Qiuzhan Zhou, Qi He, Yuzhu Chen, Xue Bao

**Affiliations:** College of Communication Engineering, Jilin University, Changchun 130022, China; heqi18@mails.jlu.edu.cn (Q.H.); chenyz18@mails.jlu.edu.cn (Y.C.); baoxue18@mails.jlu.edu.cn (X.B.)

**Keywords:** MET inlet effect, turbulence-laminar flow model, multichannel structure

## Abstract

The actual fluid form of an electrolyte in a molecular electronic converter is an important factor that causes a decrease in the accuracy of a molecular electronic transducer (MET) liquid motion sensor. To study the actual fluid morphology of an inertial electrolyte in molecular electron transducers, an inlet effect is defined according to the fluid morphology of turbulent-laminar flow, and a numerical simulation model of turbulent-laminar flow is proposed. Based on the turbulent-laminar flow model, this paper studies the variation of the inlet effect intensity when the thickness of the outermost insulating layer is 50 µm and 100 µm, respectively. Meanwhile, the changes of the inlet effect intensity and the error rate of central axial velocity field are also analyzed when the input signal intensity is different. Through the numerical experiment, it verifies that the thickness of the outermost insulating layer and the amplitude of the input signal are two important factors which can affect the inlet effect intensity and also the accuracy of the MET. Therefore, this study can provide a theoretical basis for the quantitative study on the performance optimization of a MET liquid sensor.

## 1. Introduction

Solid-state accelerometers are widely employed in many fields. The high frequency response of solid “inertia sensor” is better compared to that of the low-frequency domain [1]. The molecular electronic transducer (MET) liquid motion sensor has lower self-noise and better accuracy in the low-frequency range, making it a good choice for low-frequency applications [2,3].

In recent years, the method of tracing and analyzing characteristic parameters of electrochemical processes by numerical simulation has been used widely. The parameter characteristics of a MET can also be studied in this way. Zaitsev established a noise model and analyzed the influence of the self-noise of a sensor within the frequency range of 0.01–200 Hz [4]. Zhou studied the influence of a MET elastic film on its low-frequency performance through numerical simulations and designed elastic films suitable for different cavities to optimize MET characteristics [5]. Vadim studied the MET convective noise model, and the simulation results were basically consistent with the experimental data [6]. Ivan established a noise model of METs in the full frequency operating range to study the technical parameters and characteristics of METs [7]. Sun established two-dimensional and three-dimensional laminar flow models of the MET sensor, revealing the relationship between electrolyte velocity, concentration distribution of active ions, and current density [8]. Huang, Agafonov, and Yu reviewed METs as motion sensor and applied MET for planetary explorations [9].

In previous studies, a laminar flow model was established to study the flow signal of a single-channel electrolyte, and an ideal output signal was used for simulation; however, the impact of the MET multichannel coupling effect in practical applications was not considered [10]. The MET multichannel interaction causes irregular movements in some electrolytes at the inlet and outlet of the channel, which forms a turbulent area with unstable flow field distribution [11]. Laminar flow refers to the layered flow of electrolyte fluid. There is only relative sliding between two adjacent layers of fluid, and there is no lateral mixing between the flow layers. Turbulent flow means that the fluid no longer maintains a layered flow but may flow in all directions. Turbulence has radial velocities perpendicular to the axial direction, and confusion occurs between the various flow layers. The essential difference between laminar and turbulent flow is that laminar flow has no radial velocity, while turbulent flow has radial velocity. Because of the collision between the electrolyte in the reaction chamber and the outermost insulating layer of the sensitive chip during the movement and the small radius porous structure of the sensitive chip, the electrolyte flow mode in the channel changes to a more complex turbulent-laminar flow mode.

In previous studies, other researchers neglected the coupling between the reaction chamber and the multi-channel structure of the sensitive chip. The single channel model in the sensitive chip is usually simulated, and the electrolyte flow mode in the channel is considered as laminar flow. Based on this assumption, although the output signal and other characteristics of the sensor can be better represented, the model can’t entirely express the multi-channel coupling effect in the turbulent-laminar flow mode. Therefore, to solve this problem, a multi-channel model of MET is proposed; this model can better describe turbulent and laminar flow patterns than the single-channel model. In addition, this paper qualitatively analyzes the two main factors that cause the intensity of the inlet effect, and their impact on the accuracy of the sensor.

## 2. Mathematical Modeling

### 2.1. Geometry Model 

**Figure 1:**
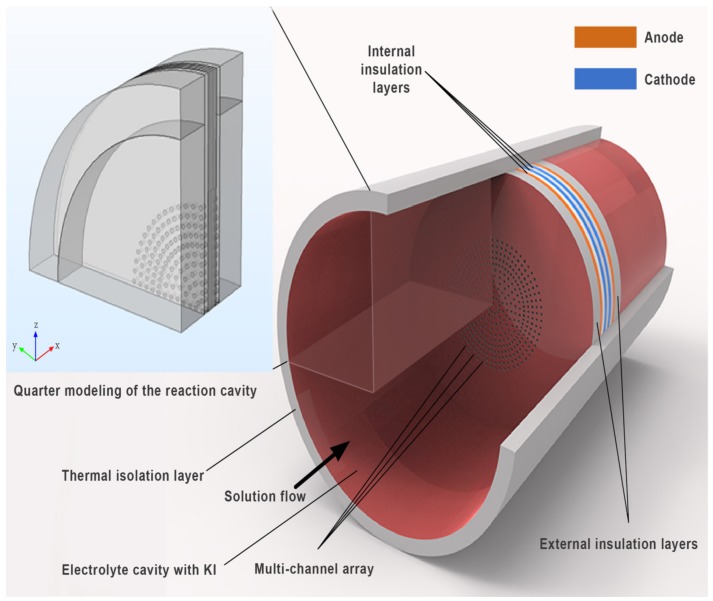
Integrated graph of the reaction cavity.

**Figure 2:**
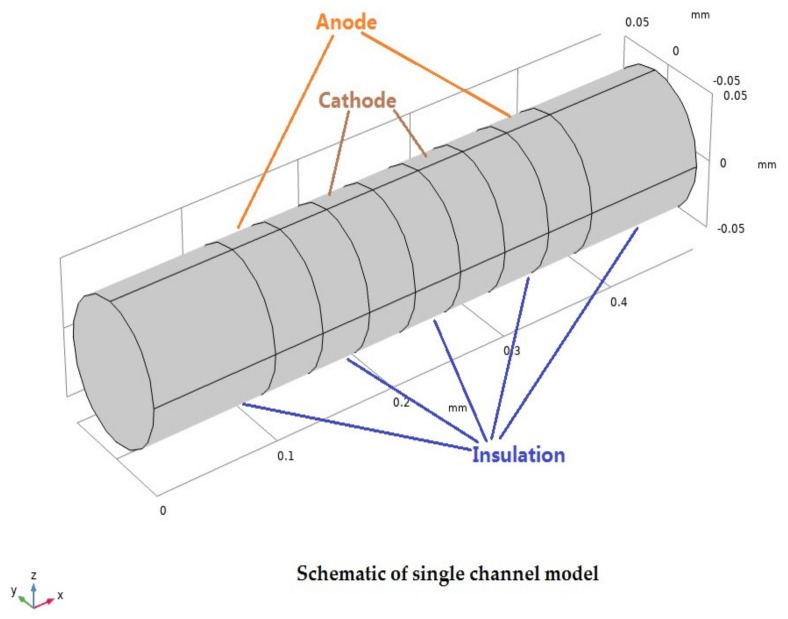
Schematic of a single channel model.

The physical structure of a MET reaction chamber is shown in Figure 1. The reaction chamber is surrounded by an insulating layer and filled with an electrolyte solution. The schematic of a single channel model is shown in Figure 2.

The sensitive chip has four platinum electrode layers (thickness: 40 µm) are arranged as per the anode - cathode - cathode - anode (ACCA) model. They are separated by three internal insulation layers (40 µm) and two outermost insulation layers (100 µm). The nine layers can be compacted into a multichannel array in the shape of a columnar channel. The channel of the sensor is circular with a radius of 0.05 mm and a spacing of 0.25 mm at the center of the channel.

As the electrolyte solution flows through the multichannel array, the solid parts of the sensor act as a barrier in addition to the channels. Obviously, the solid barrier can form an inlet effect on each channel, and therefore, the electrolyte flow in multiple channels is no longer a laminar flow, but a turbulent-laminar flow.

### 2.2. Numerical Model

We use the incompressible Navier-Stokes equations to study the electrolyte flow field in the reaction chamber [8]:

(1) $$\frac{\partial u}{\partial x}+\frac{\partial v}{\partial y}+\frac{\partial w}{\partial z}=0$$

(2) $$\frac{\partial u}{\partial t}+\frac{\partial uu}{\partial x}+\frac{\partial vu}{\partial y}+\frac{\partial wu}{\partial z}=-\frac{1}{\rho }\frac{\partial \vec{P}}{\partial x}+\frac{\mu }{\rho }\nabla^{2}u+a_{x}$$

(3) $$\frac{\partial v}{\partial t}+\frac{\partial uv}{\partial x}+\frac{\partial vv}{\partial y}+\frac{\partial wv}{\partial z}=-\frac{1}{\rho }\frac{\partial \vec{P}}{\partial y}+\frac{\mu }{\rho }\nabla^{2}v+a_{y}$$

(4) $$\frac{\partial w}{\partial t}+\frac{\partial uw}{\partial x}+\frac{\partial vw}{\partial y}+\frac{\partial ww}{\partial z}=-\frac{1}{\rho }\frac{\partial \vec{P}}{\partial z}+\frac{\mu }{\rho }\nabla^{2}w+a_{z}$$

where u, v, w represent the velocities in the X, Y, and Z directions, respectively; $a_{x}$ $a_{y}$ $a_{z}$ represent acceleration in the three directions; $\vec{P}$ represents the pressure; and *t* represents the time; the density of the electrolyte is:

(5) $$\rho =\rho_{w}+\sum_{i=1}^{k}\left\{c_{i}[B_{1i}+B_{2i}(T-273.15)+B_{3i}c_{i}]\right\}$$

and the dynamic viscosity of the electrolyte is:

(6) $$\mu =\mu_{w}\exp\{\sum_{i=1}^{k}c_{i}[A_{oi}+A_{1i}(T-273.15)+A_{2i}c_{i}+A_{3i}{\left(T-273.15\right)}^{2}]\}$$

where, $\rho_{w}$, $\mu_{w}$ are the density and viscosity of water, c is the mass fraction of electrolyte in the electrolyte, $A_{ij}$, $B_{ij}$ are the coefficients of different kinds of salt and T is the temperature of the electrolyte.

In this study, we used the Poisson equation to describe the electric potential:

(7) $$-\nabla \cdot (\varepsilon \nabla \varphi )=\rho^{\prime }$$

where ε, φ, and $\rho^{\prime }$ are the permittivity ($F\;m^{-1}$), potential (V), and charge density ($C\;m^{-3}$), respectively.

The mass transport ((8), (9)) subject to mass continuity (10)) is still described by the Nernst-Planck equation [12]:

(8) $${\vec{N}}_{i}=-D_{i}\nabla c_{i}-z_{i}m_{i}c_{i}\nabla \varphi +c_{i}\vec{u}$$

(9) $$c_{K^{+}}=c_{I_{3}^{-}}+c_{I^{-}}$$

(10) $$\frac{\partial c_{i}}{\partial t}+\nabla \cdot {\vec{N}}_{i}=R_{i}=0$$

where ${\vec{N}}_{i}$ is the flux of species i ($mol\;m^{-2}s^{-1}$); $D_{i}$ is the diffusion coefficient of species i ($m^{2}s^{-1}$), and here, we set $D_{I^{-}}=D_{K^{+}}=2.8\times 10^{-9}$ $m^{2}/s$ and $D_{I_{3}^{-}}=2.0\times 10^{-9}$ $m^{2}/s$; $c_{i}$ is the concentration of species i ($mol\;m^{-3}$); $z_{i}$ is the charge number of species i;$m_{i}$ is the mobility of species i ($m^{2}V^{-1}s^{-1}$);$\vec{u}=[u,v,w]$ is the velocity vector ($m^{}s^{-1}$); and $R_{i}$ is the mass source of species i ($mol\;m^{-3}s^{-1}$).

Because the model is highly nonlinear, it needs to be approximated by the time measurement of multiple lengths and scales. It is assumed that the electrolyte is electrically neutral in the entire process, the concentration of active ions is relatively small compared to the concentration of the background electrolyte [13,14]. Since the whole electrolyte is electrically neutral, the concentration gradient of the effectively charged ions and the current density in the electrolyte obey Ohm’s law:

(11) −∇⋅(σ∇φ)=Q

under constant conductivity:

(12) $$\sigma \approx F\sum_{i}z_{i}^{2}m_{i}c_{i}$$

Meanwhile, the concentration and velocity of electrolyte in incompressible flow are constrained by Fick’s second law:

(13) $$\frac{\partial c_{i}}{\partial t}=D_{i}\nabla^{2}c_{i}-\vec{u}\cdot \nabla c_{i}$$

where σ is conductivity ($S\;m^{-1}$), Q is charge source ($A\;m^{-3}$), and F stands for the Faraday constant.

Under the assumption of electrolyte neutrality, the abovementioned partial differential equation and Nernst-Planck equation are combined to describe the activity of ions on the electrode surface:

(14) $$2\vec{n}\cdot {\vec{N}}_{I_{3}^{-}}=-\frac{2\vec{n}\cdot {\vec{N}}_{I^{-}}}{3}=-2k_{a}c_{I_{3}^{-}}e^{\left(-\alpha F/RT)(U-\varphi -E_{0}\right)}+k_{c}c_{I^{-}}e^{\left(1-\alpha )(F/RT)(U-\varphi -E_{0}\right)}$$

where $\vec{n}$ is the charge per mole of electrons, $k_{a}=k_{c}=4\times 10^{-9}$ $m^{2}/s$ are the reaction constant representing the cathode and anode, α is the charge and discharge coefficient of the cathode reaction set at 0.5, U = 0.8 V is the potential difference between the two pairs of electrodes, and $E_{0}=0.54$ V is the equilibrium potential [15].

### 2.3. Boundary Conditions and Parameter Settings

The composition of the model electrolyte is defined as iodine-potassium iodide solution. The essence of the reaction is the mutual transformation of iodide and triiodide ions. i.e., $I_{2}+I^{-}⇌I_{3}^{-}$. Potassium ions do not participate in the reaction, and therefore, zero flow boundary conditions should be used for potassium ions on the electrode. To ensure that the simulation results converge easily, the nonslip boundary condition is applied to all solid surfaces. On the surface of the insulation layer, the electrical insulation condition is applied for the electric field and the zero-ion flow boundary condition is applied for the ion transmission. At the outlet, considering the distance from the electrode, the effect of the electric field is ignored. Initial parameter settings are listed in Table 1 [8].

In this paper, COMSOL Multiphysics, which is powerful in electrochemical simulation, is selected to conduct multi-physical field modeling and simulation analysis. The calculation of the multi-channel simulation model is completed by the following procedures. First, the author adds the Nernst-Planck equation and fluid laminar flow model in COMSOL Multiphysics and then defines the reaction cavity and sensitive element model according to the geometric model size, secondly, defines the MET parameters according to the initial parameters and boundary definition conditions. Thirdly, the electrolyte composition of MET is defined as iodine-potassium iodide, the material of the sensitive element is platinum, and the insulating material is ceramic. Finally, the mesh is divided and the electrode part is encrypted.

## 3. Simulation and Discussion

### 3.1. Evidence of Turbulence - Laminar Flow Phenomena and Multichannel Inlet/outlet Effects

**Figure 3:**
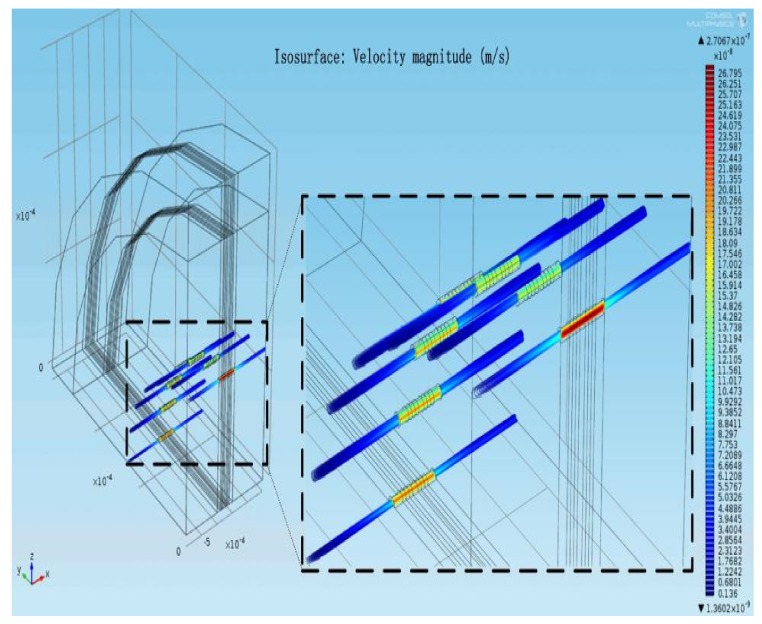
Electrolyte motion in reaction cavity at 5 s under b $a=0.01sin(\pi t)\;m/s^{2}$.

The above mathematical model was constructed by Comsol Multiphysics, a multi-physics finite element analysis software. By applying a sinusoidal signal with an acceleration of $\;a=0.01sin(\pi t)\;m/s^{2}$ to the electrolyte in the reaction chamber, the distribution of the electrolyte flow field under the model in Figure 1 can be obtained as shown in Figure 3.

.

Figure 3 shows that at the inlet and outlet of the channel structure, the kinetic energy of the electrolyte is accumulated and consumed partially. Because the electrolyte undergoes two kinds of movements with large differences in space size from the reaction chamber to the porous channel during the movement, the kinetic energy of the electrolyte will be concentrated at the inlet and outlet positions of the sensitive chip. Therefore, the particle in the electrolyte move in an irregular direction. The random movement of the electrolyte is concentrated on the inlet/outlet of the multi-channel structure of the sensitive chip, which indicates there is the inlet effect on the inlet/outlet positions of the sensitive chip in the reaction chamber.

**Figure 4:**
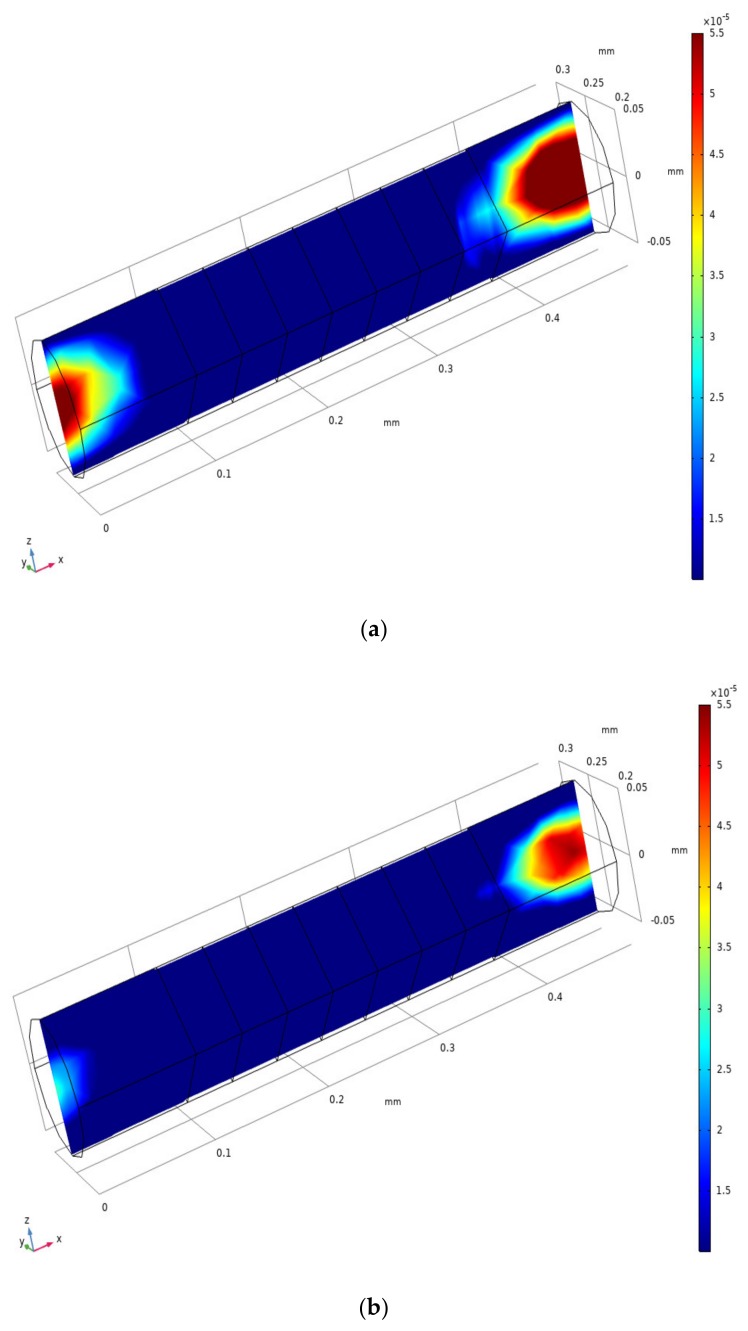
Radial velocity field distribution of the xz plane in the sensing element under (**a**) turbulent-laminar flow model and (**b**) laminar flow model.

The flow mode of the electrolyte was simulated with a new model (Figure 4a) and compared with the simulation of Sun’s laminar flow model (Figure 4b) [8,9,10,11]. As shown in Figure 4a, the radial velocity distribution gradually decreases from the inlet position, tends to zero near 90 µm, and maintains a state where the radial velocity tends to zero at 90 µm to 370 µm. At 370 µm, the radial velocity distribution increases gradually from zero. As shown in Figure 4b, the radial velocity distribution decreases gradually from the inlet position, tends to zero near 40 µm, and maintains a state where the radial velocity tends to zero at 40 µm to 400 µm. At 400 µm, the radial velocity distribution increases gradually from zero. In Figure 4a,b, the phase in which the radial velocity field tends to zero refers to the phase in which the fluid mode of the electrolyte in the channel of the sensor changes to the completely laminar flow mode. The area where the radial velocity field fluctuates at the inlet and outlet of the channel is the turbulent area. We define the change in the fluid flow state of the electrolyte that occurs at the inlet and outlet of the channel as the inlet effect. In Figure 4b, there is a turbulent region in the inlet and outlet area. Because the laminar flow model ignores the effects of channel-to-channel and channel-to-screen structure barrier of the sensitive chip. The model becomes very narrow in the turbulent region within the inlet and outlet areas. The velocity field distribution in the laminar flow model is also affected by the inlet effect. Figure 4a, the change in the electrolyte flow state from turbulent to laminar and from laminar to turbulent requires a longer distance; the variation trend of the radial velocity field in Figure 4a is smoother than that in Figure 4b. The fluid changes described at the inlet and outlet locations are more realistic. Thus, based on the above description, the turbulent-laminar flow model can better describe the inlet and outlet effect.

### 3.2. Analysis of the Inlet/outlet Effect

**Figure 5:**
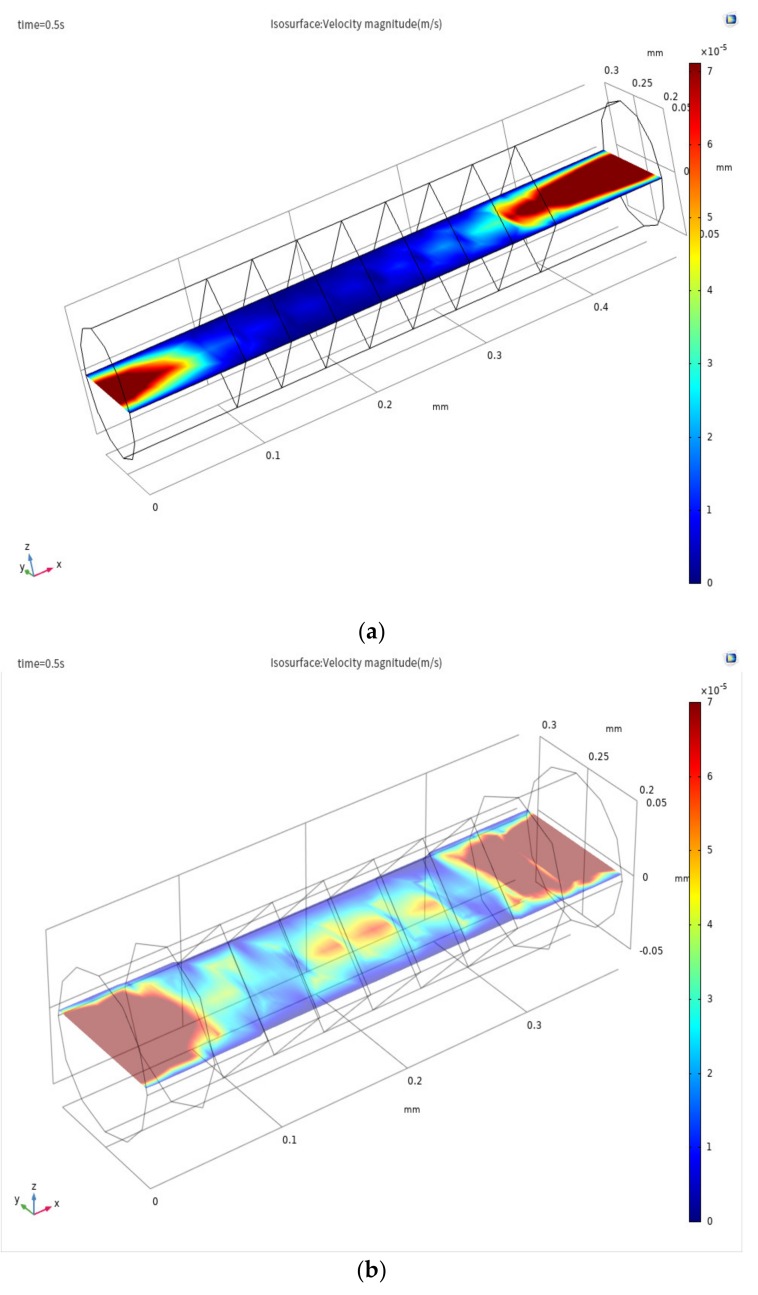
Axial velocity field distribution of the xy plane in the sensing element when the thickness of the outermost insulation layers is (**a**) 100 µm and (**b**) 50 µm.

As shown in Figure 5a, when the thickness of the outermost insulation layers is 50 μm, the intensity of the inlet effect is large, and the change in the axial velocity distribution field caused by the inlet effect reaches the electrode; the inlet effect causes a velocity field in the electrode region where the electrochemical reaction occurs; this affected the accuracy of the chip, and therefore, the output current of the electrode cannot maintain the correct outermost excitation signal. As shown in Figure 5b, when the thickness of the outermost insulation layers is 100 μm, the intensity of the inlet effect is small, and the change in the axial velocity field distribution will not spread to the vicinity of the electrode, and the effect of the inlet effect on the accuracy of the sensor is small. This shows that the longer the outermost insulation layer, the smaller is the influence of the inlet effect, and the smaller the influence of the inlet effect on the accuracy of the sensor.

**Figure 6:**
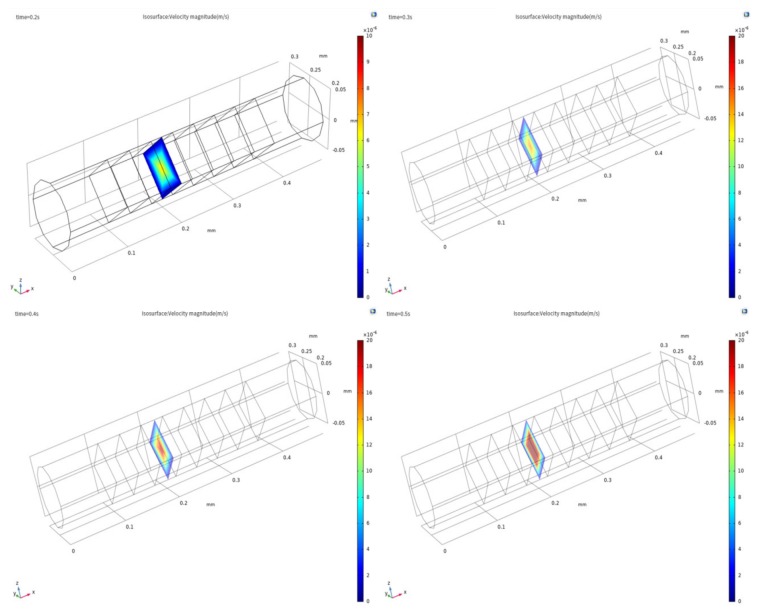
Axial velocity field distribution of the yz plane in the sensing element under sinusoidal excitation signal in the quarter cycle.

**Figure 7:**
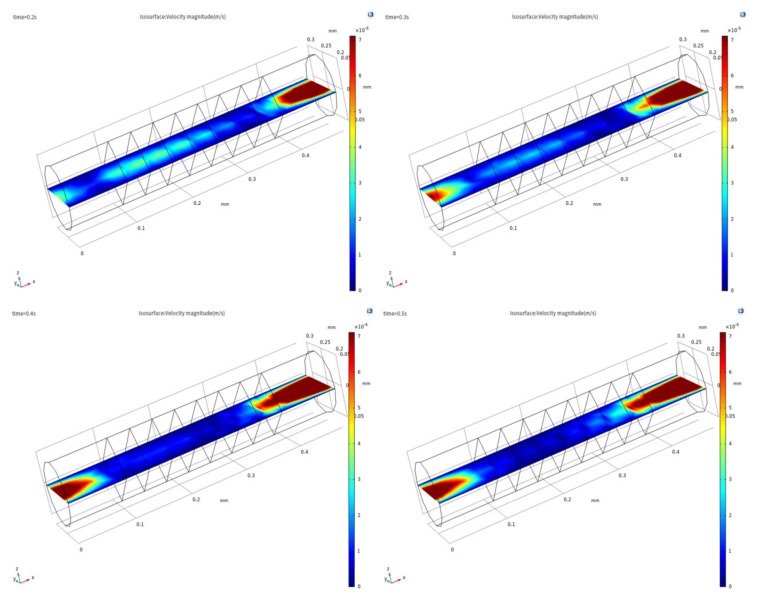
Axial velocity field distribution of the xy plane in the sensing element under sinusoidal excitation signal in the quarter cycle.

We take the velocity field distribution in the quarter cycle under the sinusoidal excitation signal as shown in Figure 6 and Figure 7. As shown in Figure 6, the amplitude of the axial velocity field increases gradually as the intensity of the excitation signal increases. As shown in Figure 7, the area affected by the inlet effect of the axial velocity field increases gradually as the intensity of the excitation signal increases, the inlet effect intensity increases gradually. This change will cause the velocity field near the electrode to become unstable, which will result in a velocity field in the electrode region to be disordered, thereby affecting the accuracy of the sensor.

To further study this influencing factor, we define a variable called error rate—

$e_{x}=\frac{v_{x}-v_{steady}}{v_{steady}}\times 100\%$—to describe the influence of the inlet effect on sensor accuracy; in this variable, $v_{x}$ is the axial velocity on the central axis in the channel, and $v_{steady}$ is an ideal stable value of the axial velocity on the central axis in the channel that is not affected by the turbulent laminar noise.

**Figure 8:**
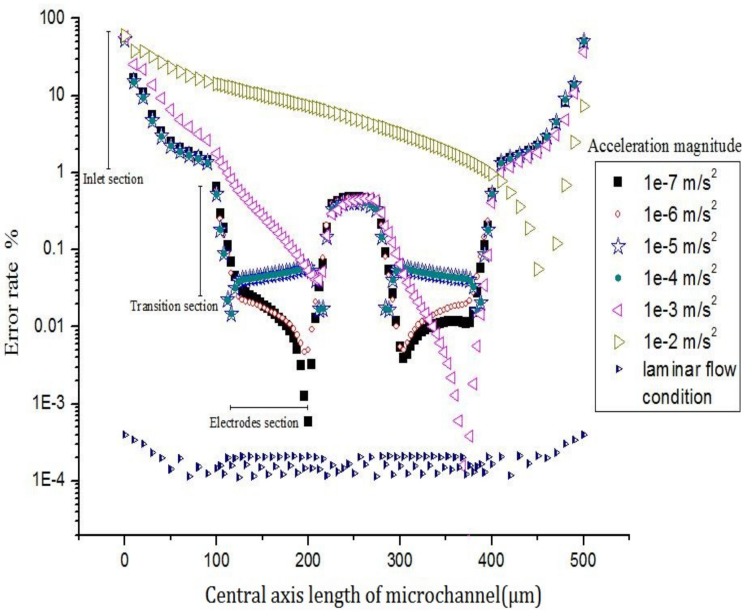
Error rate of central axis velocity in sensing element.

In Sun’s laminar flow condition, the result of the error rate is the same and the relationship between the amplitude of the input signal and the error rate is not evident. Because there is no effect of inlet effect in the model under the simple laminar flow condition, the error rate in the sensor channel is almost unchanged. The error rate is always low and can be considered as an ideal state. Further, the acceleration of the excitation signal increases with an increase in the intensity of sinusoidal excitation signal, as shown in Figure 8.

When the electrolyte in the channel of the sensitive chip moves by a small acceleration (from $a=1e^{-7}m/s^{2}$ to $a=1e^{-4}m/s^{2}$), the error rate is about 0.1%, which is in the lower area, and the axial velocity is less affected by the inlet effect. Thus, the inlet effect has a small influence on sensor accuracy.

When the electrolyte in the channel of the sensitive chip moves by a large acceleration ($a=1e^{-3}m/s^{2}$ or $a=1e^{-2}m/s^{2}$), the error rate is about 1%–10%, which is in the higher region, and the axial velocity is greatly affected by the inlet effect. Thus, the inlet effect has a big influence on sensor accuracy.

Figure 6 shows that, the greater the excitation signal strength, the greater is the influence of the inlet effect on axial velocity. In conclusion, the higher the excitation signal strength is, the higher the error rate will be. Also the greater the intensity of the inlet effect is the larger impact on sensor accuracy will be, therefore, the amplitude of the signal input is an important factor which can affect the strength of the inlet effect and the accuracy of the sensor.

The inlet effect is influenced by many factors, such as the composition of the electrolyte, and the number, size, location and shape of the holes in the sensor. In this paper, it only covers two qualitive factors for the influence of inlet effect, which are the thickness of the outermost insulation layer and the amplitude of the input signal. The other factors on the inlet effect and some various factors in a quantitative way will be our future research orientation.

## 4. Conclusions

In previous studies, a laminar flow model was used to study the morphology of electrolyte fluids, ignoring the effect of the coupling inlet effect between multichannel structures in an actual scenario. The turbulent-laminar flow model proposed in this paper can better describe this inlet effect. Compared with the single-channel laminar flow model, the real advantage of this model lies in the coupling design of the reaction chamber and sensitive element models. The simulated electrolyte fluid flow pattern is closer to the actual situation. We can rely on this model to set different MET structural parameters and boundary conditions, and apply it to MET noise research, MET elastic membrane structure research, electrolyte flow field research in the reaction chamber, etc. This model provides not only a more realistic simulation model for studying MET performance and also helps to optimize the performance of MET and the configuration of achieving the optimal performance of MET sensors for different fields and application environments. Therefore, the model in this paper can broaden the application field of MET electrochemical sensors.

Through a numerical simulation, the actual fluid morphology of an electrolyte was studied qualitatively, and the existence of the inlet effect was proved. Simultaneously, it was found that the thickness of the outermost insulation layer and the amplitude of the input signal are the two factors that affect the intensity of the inlet effect and the accuracy of the sensor. The longer the thickness of the outermost insulation layer, the smaller is the influence of the inlet effect, and smaller is the influence on the accuracy of the sensor. The larger the amplitude of the input signal, the greater is the impact of the inlet effect, and greater is the impact on the accuracy of the sensor. In this paper, two factors that affected the intensity of the inlet effect and the accuracy of the sensor are studied qualitatively; however, no method was proposed to improve the accuracy of MET using this effect in practical applications.

In future studies, we will optimize the structural layout of the sensor elements in the reaction chamber through quantitative research on the thickness of the outermost insulation layer. Simultaneously, we will study the amplitude of the input signal using vibration-level calibration, which corresponds to different application scenarios and vibration signals. Sensors with different vibration-level calibrations are used to achieve the maximum suppression of the fluctuation which is caused by the electrolyte velocity field change. It is expected that through these studies, the effectiveness of MET liquid sensor can be further optimized for better application in more fields.

## Figures and Tables

**Table 1:** Parameters of simulation model.

Characteristic Parameters	Symbol	Value
Electrolyte temperature	T	300 K
Ion mobility	$D_{I^{-}},D_{K^{+}}$	$2.8\times 10^{-9}\;m^{2}/s$
	$D_{I_{3}^{-}}$	$2.0\times 10^{-9}\;m^{2}/s$
Electrode reaction constant	$k_{a},k_{c}$	$4\times 10^{-9}\;m^{2}/s$
Equilibrium potential	$E_{0}$	0.54 V
The density of water	$\rho_{w}$	$1000\;kg/m^{3}$
Viscosity of water	$\mu_{w}$	$1.0\times 10^{-3}\;Pa\cdot s$
The density of electrolyte	ρ	$1473\;kg/m^{3}$
Viscosity of electrolyte	μ	$1.4\times 10^{-3}\;Pa\cdot s$
Electrolyte temperature	T	300 K
Ion mobility	$D_{I^{-}},D_{K^{+}}$	$2.8\times 10^{-9}\;m^{2}/s$
